# Promotive and Preventive Factors Influencing Adapting of Resin Infiltration: A Qualitative Study

**DOI:** 10.1155/ijod/1757672

**Published:** 2026-07-22

**Authors:** Päivi Havela, Tarja Tanner, Taina Kankaala, Vuokko Anttonen, Marja-Liisa Laitala

**Affiliations:** ^1^ Research Unit of Population Health, Faculty of Medicine, University of Oulu, Oulu, Finland, oulu.fi; ^2^ Medical Research Center Oulu and Oulu University Hospital, Oulu, Finland

**Keywords:** caries prevention, minimal invasive, qualitative study, resin infiltration

## Abstract

**Objectives:**

The aim of this qualitative study was to identify Finnish dentists’ perspectives on resin infiltration (RI). The specific targets of interest were promotive and preventive factors influencing the implementation of RI.

**Methods:**

A Webropol‐based electronic questionnaire was sent to all members of the Finnish Dental Association (FDA) via a regular newsletter in spring 2022. The questionnaire was resent in September 2022 to the supervisors of dentists for further distribution. In addition to the structured questions, respondents were allowed to provide free‐form comments on RI. All open‐ended comments (*n* = 59) were derived, analyzed, and grouped using inductive content analysis to create sub‐ and main categories.

**Results:**

In the content analysis, four main categories were obtained: The first main category, “Competence,” was divided into *Competence-maintaining skills*, *Attitude*, and *Demanding technique* generic categories. The second main category, “Resources,” was divided into *Costs* and *Human resources* generic categories. The third main category, “Caries control” included one generic category, *Timing of resin infiltration*. Division of tasks was the generic category of the fourth main category, “*Working duties*.” The most frequent barriers for the use of RI were limited resources—both monetary and human resources—and lack of competence.

**Conclusions:**

Finnish dentists seem to be willing to adopt new procedures. New treatments such as RI require adequate resources, and both theoretical and practical training.

## 1. Introduction

Approximately 3.5 billion individuals are affected by oral diseases, and according to the Global Burden of Diseases 2019, untreated caries in permanent teeth is the most common noncommunicable disease worldwide [1]. In modern caries management approaches, the aim is to arrest the progress of active initial lesions, that is, fluoride applications, fissure sealants on occlusal surfaces, and resin infiltration (RI) [2]. The arresting of carious lesions leads to a gradual increase in mineral content compared to active lesions, beginning with a notable rise in mineral density on the outermost surface of the enamel [3].

The RI technique was developed in the early 2000s to provide a method specifically aimed at arresting the progression of initial caries lesions in proximal tooth surfaces (ICDAS scores 2 or 3) [4, 5]. RI is claimed to provide numerous advantages, such as preserving tooth structure, improving the esthetics in white spot lesions, preventing caries progression, filling micropores in the lesion body, delaying the need for restoration, reducing recurrent caries, and eliminating inflammation [6]. The effectiveness of RI in halting proximal noncavitated caries lesions has been broadly reported [7–10]. According to recent systematic reviews, RI has demonstrated notable effectiveness among minimally invasive approaches for managing carious lesions in pediatric dentistry [11, 12].

To perform RI successfully, diligence and adequate operator competence are essential [13]. Dental professionals have reported that the RI procedure is somewhat laborious and time‐consuming; the main challenges were tooth separation, protecting soft tissues, and maintaining the dryness of the operated teeth [13–15]. From the point of view of patients, RI was generally well tolerated among adults and adolescents [14], but among children, RI was associated with discomfort despite local anesthesia [16].

Several factors have been identified as influencing dentists’ behavior in controlling caries. These include the dentist’s age as well as insufficient knowledge regarding caries pathogenesis and caries control methods. Additionally, guidelines, peer influence, professional identity, and environmental factors, such as financial or regulatory aspects, play a role in the use of RI. To comprehend how these factors impact decision‐making in a particular context and to develop interventions for modifying dentists’ behavior, qualitative studies are necessary [17, 18].

Previously, we found a positive attitude in our semistructured questionnaire‐based survey towards RI by those clinicians who were actively doing it. RI was considered a useful minimally invasive measure for arresting caries and also suitable for use by dental hygienists [19].

The aim of this qualitative study using inductive content analysis was to identify the perspectives of Finnish dentists on RI with qualitative research methods. Specifically, factors promoting and inhibiting the use of RI were targets of interest.

## 2. Methods

### 2.1. Study Population

The study population comprised members of the Finnish Dental Association (FDA). A link to an electronic Webropol‐based questionnaire was sent to all members of the FDA via the regular newsletter (*n* = 6687 in 2022) in April 2022 and resent after a month. In September 2022, the same link to the questionnaire was sent to the managers of the largest private dental companies in Finland, to the Finnish Student Health Services, and to the managers in public oral health care to be delivered to all dentists. Both the Finnish and Swedish versions of the questionnaire were available. The questionnaire was finally closed in October 2022. Besides nine questions with options for answers, it was possible for the participants to provide additional comments on RI in an open‐ended question. As background factors, years since graduating (less than 5 years/5–10 years/more than 10 years), dental school (Helsinki/Turku/Oulu/Eastern Finland), and information sources concerning RI (during undergraduate studies/after graduate education/introduced by a colleague/from journals related to the field/elsewhere, where?) were assessed [19].

### 2.2. Qualitative Data Analysis

The first author read the responses to the open‐ended question several times to identify and delineate main issues related to the research themes. All responses were then individually reviewed multiple times by four members of the research group, who brought different perspectives to the discussions (an experienced professional in qualitative research and three dentists familiar with the academic/organizational/clinical practices on the topic). By repeatedly reading the data, the aim was to uncover the meaning and achieve a deep understanding of the issue and ensure accurate interpretation. To achieve the agreement, research group members discussed together several times in Teams meetings.

Key phrases used by the respondents were highlighted, with attention given to their emphasis and meaning. The responses were reviewed again, this time focusing on identifying patterns of similarities and differences. After becoming familiar with the raw data, similar responses were condensed into codes. These condensed expressions were then grouped according to content similarity, further abstracted into subcategories and main categories, and finally labeled based on their content. In the analysis, no software or AI was used.

The trustworthiness of the findings was achieved: the responses were analyzed and interpreted by four members of the research group, ensuring the triangulation. The coding and categorization of the data were transparent, and the authors were aware of the importance of reflexivity. Furthermore, the authenticity and transparency of the analysis were demonstrated with several quotations extracted from the original data.

### 2.3. Ethical Considerations

Participation was voluntary, and respondents were not financially compensated. Webropol maintained anonymity, ensuring that the authors could not access respondents’ names at any phase during the survey. According to Finnish legislation, an ethical review by the Regional Medical Research Ethics Committee was not needed, as the survey did not affect participants’ physical or psychological integrity [20].

## 3. Results

Altogether, 416 dentists participated in the survey, and of those, 59 gave open‐ended comments. Half of the respondents (*n* = 30) who provided open‐ended comments had graduated more than 10 years earlier, and a third (*n* = 22) less than 5 years earlier. Two‐thirds (*n* = 42) of the respondents had graduated from the University of Oulu or the University of Helsinki, and the rest (*n* = 17) from the other two dental schools in Finland (the University of Turku and the University of Eastern Finland) or outside Finland.

Of the respondents, one‐third (*n* = 24) reported having received information on RI during their undergraduate studies. Continuing education courses and dental journals on the topic were also mentioned by a third (*n* = 20) of the respondents. Other sources mentioned were the internet and YouTube.

**Figure 1:**
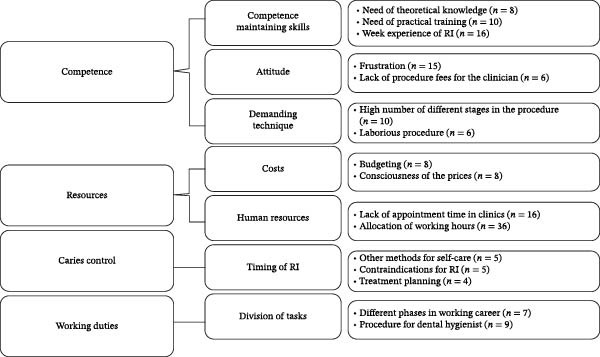
Four main categories, and their generic categories and subcategories derived through content analysis from Finnish dentists’ open‐ended responses regarding the use of resin infiltration. The numbers indicate the frequencies of the responses in each subcategory.

In the analysis process, free‐form comments on the use of RI were divided into four main categories: (1) competence, (2) resources, (3) caries control, and (4) working duties. These four categories included 1–3 generic categories (Figure 1).

### 3.1. Competence

Respondents commented that being able to perform RI in real‐life situations; it is more than just theoretically knowing something. The procedure was mentioned to be laborious for a clinician, and the possibility for practical training before adapting it was highlighted. Most of the respondents had not learned the RI technique during their undergraduate studies and were not familiar with it or reported only a slight experience. Some respondents questioned the scientific evidence, considering it weak or nonexistent, while others were not convinced of the effectiveness of RI.

Competence was divided into three generic categories: (1a) competence‐maintaining skills, (1b) attitude, and (1c) demanding technique. All three also included subcategories (Figure 1).

Competence‐maintaining skills were further divided into three subcategories: the need for theoretical knowledge, the need for practical training, and insufficient experience with RI. Theoretical knowledge included statements emphasizing the need for better scientific evidence and information about RI. Also, training for dental hygienists was mentioned.

*“So far*, *the challenge has been a lack of knowledge.”*

*“More evidence is needed.”*

The need for practical training and insufficient experience were common; respondents wanted to practice RI step‐by‐step, hands‐on, and with guidance. Also, they were not familiar with the equipment required for RI.

*“In order to learn, supervision would be important when you try the method for the first time.”*

*“Requires practical training before including RI in your own toolbox.”*

*“I saw only a short video during my studies.”*

The attitude was divided into two subcategories: frustration and lack of financial compensation for the clinician. Frustration appeared in comments where initial caries lesions were left without any intervention, and eventually, restorative treatment was necessary when the lesion reached the cavitation stage. On the other hand, measures aimed at arresting caries lesions were not found to be convincing. Some responses even referred to RI as a “nonsense therapy.” Respondents indicated that clinicians should receive extra compensation for performing RI.

*“Education and the union* [meaning FDA] *encourage nonsense therapy.”*

*“Compensate* (*the clinician*) *for performing RI rather than filling*, *especially in children and adolescence.”*

The demanding technique of RI was one of the three generic categories of competence. The high number of different stages/multiple phases in the RI procedure was perceived as laborious. One respondent wondered how painful RI measures are for a patient. One respondent thought that RI done proximally was too demanding for a dental hygienist.

*“RI requires separation with orthodontic rubber rings between molars.”*

*“It takes times to tinker with it* (*rubber dam*).*”*

### 3.2. Resources

The prices of RI materials were considered to be too high to adopt RI in routine patient care. High costs were mentioned to be the reason for administrative guidelines and decisions that prevent the implementation of RI. Respondents reported that they already have too much work, their appointment schedules are full, and no new appointments are available. Some of the respondents commented that restorative treatments were more important than new caries‐control methods.

Based on the comments, resources were divided into two generic categories: (2a) costs and (2b) human resources (Figure 1). Material costs were found to be high, and the purchase of RI equipment for clinics was not allowed.

*“The bosses won’t let us order RI because it costs too much.”*

*“High price of the product limits its usage.”*

*“I would use it if public/primary health care could afford to buy it.”*

RI was not prioritized due to the limited human resources in the clinics. A lack of dental staff prevented treatments such as RI, which were considered unnecessary.

*“We do not even have enough time for restorative treatments.”*

*“Within public health care*, *there’s no time for treatments for caries arrest unless a separate appointment is booked.”*

*“There are not resources even for basic dental care for children and the elderly.”*

*“So far*, *the main issue has been a lack of treatment resources.”*

### 3.3. Caries Control

Prevention and control of caries were found to be important and one of the main tasks in oral health care, although some cynical attitudes were also raised. The importance of oral self‐care was emphasized; respondents commented that RI is not suitable for patients who are not interested in taking care of their oral health. Some of the respondents commented that traditional prevention methods such as fluoride varnish and dietary counseling are effective enough, and no additional methods are necessary. Positive comments were related to optimal timing for prevention, and minimally invasive interventions instead of extensive restorative treatments were preferred.

In caries control, the generic category of timing of RI was divided into three subcategories: other preventive measures for self‐care, contraindications for RI, and treatment planning (Figure 1). Traditional methods, such as toothbrushing and interdental flossing, were emphasized as crucial preventive measures to prevent and control caries.

*“Teaching and motivating patients for home care before the next attempt.”*

The contraindications for RI were patients’ high risk of developing new caries lesions, low level of self‐care, and existing cavitated caries lesions.

*“Some patients do not even brush their teeth.”*

*“I mainly see patients with really extensive caries lesions, which can no longer be arrested by any means other than restorative treatment, extensive restorations and removals, etc.”*

Creating an individualized treatment plan for the patient was part of caries treatment.

*“But in reality, we need skilled clinicians who carry out timely check-ups and create comprehensive treatment plans.”*

*“RI is a useful extra in the toolbox.”*

### 3.4. Working Duties

Some of the respondents stated that due to their specialty or field of interest, they do not perform RI. Preventive and minimally invasive measures were seen as a part of the core responsibilities of dental hygienists. Especially RI on smooth tooth surfaces was considered to be a task for hygienists. The generic category of working duties was defined as the division of tasks inside the dental team, including the responsibilities of dentists, hygienists, and dental nurses. Two subcategories were found: different phases of one’s working career and the role of dental hygienists. Different career phases included comments by respondents who did not have children or adolescents as patients at all, or did not have patients with cariological problems, and dentists who did only surgical or prosthetic treatments (Figure 1).

As a clinical measure, RI was seen as part of the working duties of dental hygienists inside the dental team.

*“At least dental hygienists could learn the technique.”*

*“The idea is to train dental hygienists.”*

*“A procedure that falls within the scope of dental hygienists’ competence.”*

*“Due to limited resources*, *it’s most cost-effective and convenient for the dental hygienist to carry it out.”*

## 4. Discussion

The sufficient and relatively high number of free‐form comments suggests a high level of interest among Finnish dentists in the RI procedure. The survey revealed obstacles for adapting RI, but positive opinions and experiences were also reported. The most frequent barriers to the use of RI were limited resources—both monetary and human resources—and lack of competence. Finnish dentists consider RI to be an important modern complement to traditional methods, such as self‐care guidance and fluoride treatments.

Encouragingly, the RI procedure had been attempted, and the plan was to continue with suitable patients. Dentists demonstrate a sustained commitment to professional development, actively seeking to learn and integrate new practices, and continue to be willing to engage in continuing education throughout their careers. Insufficient competence, including both theoretical knowledge and practical skills, was seen as a major barrier to adapting RI. The teaching of the RI technique in Finnish dental schools has been included in the curricula for only a relatively short time, ~10 years, and therefore, this finding can be at least partly expected. According to Schneider [21], competence is the demonstrated capacity to apply relevant knowledge, skills, and attitudes to perform tasks or roles effectively and in accordance with defined standards within a specific context. It includes both technical proficiency and the ability to adapt to real‐world demands [21]. Currently, Finnish dental students get comprehensive theoretical education in RI during their undergraduate studies, and after theoretical studies, they are eligible to begin clinical practice with patients in teaching clinics. It can be presumed that awareness and also competence will improve in the future. However, the adoption of a new measure requires repetition; hence, theoretical knowledge alone at the beginning of a career is not enough; practical training and continuing education are also important.

A lack of knowledge and competence also affects attitudes. A suspicious or even negative attitude prevents the adoption of a new model of action. Frustration associated with the effectiveness of caries preventive methods is a worrying phenomenon and an issue of considerable concern within health care practice. According to Bandura [22] and later also Miller [23], effective ways to change attitudes include creating a sense of conflict, using clear and emotionally engaging communication, leveraging role models, supporting autonomy and competence, and making small, gradual changes in behavior. By improving both theoretical understanding and hands‐on training, the perceived complexity of the method can be reduced [22, 23].

The responses highlighted a lack of human resources or limited budgets in health care organizations, and they were the most frequently mentioned obstacles to using RI. The high price of the material, often combined with uncertainty about its effectiveness, was perceived as a barrier to RI. In both public and private health care, budgets are based on strict financial frameworks, and tight budgets limit the selection of procedures and seem to hinder the adoption of RI. The material costs of RI compared to traditional fluoride products can be considered high but still at the same level or even lower when compared to the costs of restorative treatments, not to mention the costs in the long run. Minimal interventions—such as the application of sealants and RI—have demonstrated their efficacy in arresting the caries process and preventing further lesion development [24]. Preventive and noninvasive or minimally invasive treatment protocols prior to the implementation of conventional invasive restorative procedures are a standard of contemporary good clinical practice [24]. Finnish Current Care Guidelines for clinical practice (Caries Control) [25] and ICCMS guidelines [26] emphasize noninvasive and tissue‐saving methods, and in caries management, fissure sealants and RI are recommended.

According to Linden et al. [27], nearly half of Finnish dentists’ working hours are still devoted to invasive restorative procedures for dental caries. Unfortunately, the shortage of dental staff seems to have resulted in the prioritization of tasks and the focus on acute care instead of prevention. In Finland, the salary of dentists in public health care comprises a fixed monthly salary and fees for certain measures, for example, fillings. At the time of the survey, no separate fee was received for RI, in contrast to other cariology measures. The lack of monetary compensation was an obvious reason for the negative attitude of the respondents. Despite the indisputable scientific evidence supporting the effectiveness of RI in arresting caries [7–12], some respondents did not consider the evidence strong enough to justify the procedure. RI is recommended by national clinical guidelines [25]; however, guidelines do not implement themselves. They are often underused after dissemination, and implementation efforts typically result in only modest improvements [28]. Deep‐rooted attitudes that are resistant to research may contribute to the continued use of routine, nonevidence‐based practices [29]. Administrative support and positive attitude of supervisors and organizations are essential; for example, the possibility of continuous education and access to scientific journals should be assured. Further, promoting clinical research in general practice might raise the attitude towards research and decrease the research‐to‐practice gap. If some resources could be targeted to provide adequate further training of RI, clinicians could adopt new methods more easily.

To our knowledge, research on patients’ experiences or perceptions of RI is vague. A 1‐year follow‐up study by Zotti et al. [30] examined patients’ perceptions of the treatment of fluorotic teeth. During RI treatment, tooth sensitivity was not reported by any of the participants, but after treatment, mild sensitivity disappearing within 72 h was stated. All participants viewed the duration of the procedure positively [30].

Division of tasks between dental hygienists and dentists could lighten the workload of the dentists and thereby reduce costs. The role of dental hygienists in the dental care team has been established, and with proper training, hygienists can implement new practices. The RI procedure was thought to be suitable as a task for hygienists, especially in cases involving smooth tooth surfaces. Recently, in a qualitative study by Kerälä et al. [15], the willingness of Finnish dental hygienists to perform RI was assessed. It found that including RI into the core curriculum of dental hygienist education may enhance their preparedness and competence to perform RI [15].

Compared to quantitative research, a qualitative study design can provide a deeper insight into the issue being studied, which is the main strength of this study. Content analysis can be viewed as a dialog between the researcher and the data. Here, no prior knowledge of the subject was available, and thus, inductive analysis of the data was carried out. One of the main strengths of this study is expertise not only in dentistry but also in qualitative studies. Experts in their own field can search for and further interpret—possibly without noticing it—elements and themes which confirm their former opinions and knowledge. Here, an expert in qualitative analysis offered valuable diversity when categorizations were done and discussed.

It can be speculated that there is a risk of bias, as respondents who provided comments might differ systematically from those who did not. This could not be totally avoided in the present study; however, the professional profile of the respondents varied in terms of place of undergraduate studies and work experience. When interpreting content analysis, one must also take into account the possibility of social desirability, which potentially arises from being interviewed face‐to‐face and may skew the answers to be overly positive [31]. The risk of overly positive comments was most probably lower with an anonymous questionnaire. Although all comments were included in the analysis, the interpretation of the results has to be drawn with caution. Targeting the survey only to dentists can be considered a limitation, and in the future, it would be important to invite dental hygienists to participate in the survey. It would also be interesting and important to repeat the survey to determine whether the awareness and use of RI in clinical practice have increased.

High‐quality research is essential for the development of dentistry and the establishment of new procedures for clinical work. Adopting a new way of working requires up‐to‐date competence in both theoretical and practical training. Our findings provide insight into how dentists’ perceptions of RI might be changed and how they could be encouraged to adopt it in their clinical practice.

## 5. Conclusions

Finnish dentists seem to be ready for new procedures such as RI, but adequate training and sufficient resources are necessary.

## Author Contributions

**Päivi Havela:** conceptualization, data analysis, writing – original draft, review and editing. **Tarja Tanner, Vuokko Anttonen, and Marja-Liisa Laitala:** conceptualization, data analysis, writing – review and editing. **Taina Kankaala:** writing – review and editing.

## Acknowledgments

We gratefully acknowledge the late Professor Pirjo Kaakinen for her valuable assistance and support in the field of qualitative research and inductive content analysis.

## Funding

The authors received no specific funding for this work. Open access publishing facilitated by Oulun yliopisto, as part of the Wiley ‐ FinELib agreement.

## Disclosure

All authors have read and approved the final version of the manuscript. The corresponding author had full access to all of the data in this study and takes complete responsibility for the integrity of the data and the accuracy of the data analysis.

## Conflicts of Interest

The authors declare no conflicts of interest.

## Data Availability

The data that support the findings of this study are available from the corresponding author upon reasonable request.
